# Investigations on the Influence of Collagen Type on Physicochemical Properties of PVP/PVA Composites Enriched with Hydroxyapatite Developed for Biomedical Applications

**DOI:** 10.3390/ma15010037

**Published:** 2021-12-21

**Authors:** Magdalena Głąb, Anna Drabczyk, Sonia Kudłacik-Kramarczyk, Magdalena Kędzierska, Agnieszka Tomala, Agnieszka Sobczak-Kupiec, Dariusz Mierzwiński, Bożena Tyliszczak

**Affiliations:** 1 Department of Materials Science, Faculty of Materials Engineering and Physics, Cracow University of Technology, 37 Jana Pawła II Av., 31-864 Krakow, Poland; agnieszka.tomala@pk.edu.pl (A.T.); agnieszka.sobczak-kupiec@pk.edu.pl (A.S.-K.); dariusz.mierzwinski@pk.edu.pl (D.M.); bozena.tyliszczak@pk.edu.pl (B.T.); 2 Department of Chemotherapy, Medical University of Lodz, WWCOiT Copernicus Hospital, 90-001 Lodz, Poland; kameleonmagda6@gmail.com

**Keywords:** polymer-ceramic composites, hydroxyapatite, collagen, swelling capacity, surface roughness, wettability, surface free energy, tissue engineering

## Abstract

Nowadays, a great attention is directed into development of innovative multifunctional composites which may support bone tissue regeneration. This may be achieved by combining collagen and hydroxyapatite showing bioactivity, osteoconductivity and osteoinductivity with such biocompatible polymers as polyvinylpyrrolidone (PVP) and poly(vinyl alcohol) (PVA). Here PVA/PVP-based composites modified with hydroxyapatite (HAp, 10 wt.%) and collagen (30 wt.%) were obtained via UV radiation while two types of collagen were used (fish and bovine) and crosslinking agents differing in the average molecular weight. Next, their chemical structure was characterized using Fourier transform infrared (FT-IR) spectroscopy, roughness of their surfaces was determined using a stylus contact profilometer while their wettability was evaluated by a sessile drop method followed by the measurements of their surface free energy. Subsequently, swelling properties of composites were verified in simulated physiological liquids as well as the behavior of composites in these liquids by pH measurements. It was proved that collagen-modified composites showed higher swelling ability (even 25% more) compared to unmodified ones, surface roughness, biocompatibility towards simulated physiological liquids and hydrophilicity (contact angles lower than 90°). Considering physicochemical properties of developed materials and a possibility of the preparation of their various shapes and sizes, it may be concluded that developed materials showed great application potential for biomedical use, e.g., as materials filling bone defects supporting their treatments and promoting bone tissue regeneration due to the presence of hydroxyapatite with osteoinductive and osteoconductive properties.

## 1. Introduction

The development of the medical sciences taking shape over the years had a huge impact on the extension of the average human life expectancy. This is undoubtedly a great success but simultaneously a big challenge related to the necessity of the search for effective therapies of treatments of diseases that often accompany the aging of organisms including mainly diseases of the skeletal system [1]. Importantly, a development of the so-called civilization diseases such as e.g., cardiovascular diseases, cancers or osteoporosis is also a problematic issue [2,3]. In the case of osteoporosis, the risk of bone fractures increases significantly as a result of a decrease in their mechanical resistance [4,5]. The patients suffering from this ailment often experience very serious injuries, which in the majority of cases require surgical intervention [6]. Thus, investigations on the development of novel bioactive composite materials that might support the treatment of bone defects and promote faster bone regeneration are very meaningful and constitute one of the biggest challenges of contemporary medicine.

Recently, one of the most popular research topics seems to be studies on the development of composite materials containing a ceramic phase [7,8,9]. The substance which is the most frequently used for this purpose is hydroxyapatite (HAp) which constitutes a mineral component of bones [10]. Considering its bioactivity [11,12], biocompatibility [13,14,15], osteoinductivity [16,17] and osteoconductivity [18,19], this substance has a key meaning in preparation of materials designed for bone tissue regeneration. HAp is widely used in bone substitute materials [20,21], in tissue engineering [22,23] and as a drug carrier [24,25].

Composite materials based on the combination of biodegradable synthetic and natural polymers and modified additionally with hydroxyapatite were described by Gordienko et al. The composites containing biologically active substance were analyzed in detail which allowed to conclude e.g., that the developed materials showed no cytotoxicity during in vivo experiments [26]. Next, Shirdar et al. proposed composites based on poly(methyl methacrylate) and modified with hydroxyapatite nanofibers and magnesium phosphate-based two-dimensional nanosheets. The statistical analysis performed indicated the significant impact of both modifiers on the improvement of the compressive strength of developed materials and, importantly, higher viability of cells was reported in the presence of such modified composites compared to unmodified materials [27].

Other interesting studies were described by Liu et al. Here, the main attention was focused on biodegradable composites based on poly(ε-caprolactone) (PCL) and HAp nanoparticles. The materials were prepared via 3D printing. It was observed during in vivo biological analyses that developed scaffolds based on PCL and HAp provided adequate adhesion and penetration of bone cells which, in turn, allowed bone tissue to regenerate [28].

Next, Rodzeń et al. also presented an innovative composite material designed for bone regeneration. As a base material, polyetheretherketone (PEEK) modified with HAp (30 wt.%) was used. In order to obtain a composite, one of the 3D printing methods, i.e., fused filament fabrication, was employed. It was proved that surfaces of developed PEEK/HAp composites supported adhesion and growth of U-2 OS osteoblast-like cells [29]. In other work, composite scaffolds based on HAp and bioglass obtained using a hydrothermal method were characterized. Performed in vitro biological studies confirmed that developed materials showed no cytotoxicity while their biological performance was significantly higher than in the case of unmodified scaffold [30].

Despite numerous studies on the development of innovative composite materials intended for bone tissue regeneration, the methods leading to preparation of such composites still have some limitations or require a lot of work, reagents and are time-consuming. Thus the main purpose of the research was to prepare and characterize composite materials with a wide application potential in regenerative medicine. The potential of developed materials lies in their quick and simple synthesis methodology and an interesting composition in viewpoint of their potential biomedical use which may affect bone regeneration process. Furthermore, the methodology applied gives an opportunity to synthesize composites of various shapes and sizes tailored to a given bone defect.

Furthermore, the presence of collagen of fish or bovine origin increases the innovativeness of the developed composite materials. Collagen is a main component of extracellular matrix which makes it an interesting biopolymer for applications in tissue engineering and regeneration medicine [31,32].

Collagen-based materials show interesting properties due to their good biocompatibility, ability to promote cell proliferation and adhesion, and non-toxicity. Moreover, collagen exhibits hemostatic properties, and provides the reconstructed tissues with an adequate elasticity [33]. In regenerative processes, collagen-based biomatrix imitates scaffolds that fulfill structural and mechanical functions thus favoring the reconstruction of damaged organs and tissues. Importantly, the introduction of collagen into composite biomaterials increases the effectiveness of such materials in regenerative processes, their ability to combine with growth factors, and also enables intracellular transmission and stabilization of cellular components due to which such composite plays a role of a physical structure supporting the regeneration processes [34,35].

The measurement methodology has been chosen so as to verify these composites’ usefulness and evaluate their potential for providing adequate conditions for such processes as e.g., cell proliferation. That is why such properties of developed materials were investigated as wettability (it was demonstrated that hydrophilic surfaces favor the bone growth process) or roughness. Materials’ roughness affects the interactions between such an implant and the newly formed tissue. This, in turn, has a crucial meaning in viewpoint of potential cell proliferation, and so the healing and regeneration processes.

Thus, this work examined a series of composites based on synthetic polymers such as polyvinylpyrrolidone (PVP) and poly(vinyl alcohol) and, importantly, containing also a ceramic phase (hydroxyapatite) and an additional modifying agent—collagen, while two series were prepared using two type of collagens, i.e., fish and bovine. Developed composites were subsequently analyzed in detail while the main attention was paid to verifying the impact of the type of collagen and the average molecular weight of the crosslinking agent used during the synthesis of composite materials on their physicochemical properties. For this purpose, such analyses as Fourier transform infrared (FT-IR) spectroscopy as well as swelling measurements and incubation in simulated physiological liquids were performed. Moreover, the roughness of prepared materials and the topography of their surfaces were also investigated.

## 2. Materials and Methods

### 2.1. Materials

Ammonium phosphate monobasic (NH_4_H_2_PO_4_, ACS reagent, ≥98%), calcium nitrate tetrahydrate (Ca(NO_3_)_2_·4H_2_O, ACS reagent, 99%) and ammonia water (NH_4_OH, 25%) were used to obtain hydroxyapatite (HAp). Next, poly(vinyl alcohol) (PVA, crystalline powder, 87–89% hydrolyzed, M_w_ 13,000–23,000), polyvinylpyrrolidone (PVP, powder, average mol wt. 10,000), diacrylate poly(ethylene glycol) (crosslinking agent, PEGDA, average molecular weight M_n_ = 700 g/mol and M_n_ = 575 g/mol) and 2-hydroxy-2-methylpropiophenone (photoinitiator, 97%, d = 1.077 g/mL) were applied during the preparation of composite materials. All mentioned reagents were purchased from Sigma Aldrich (Saint Louis, MO, USA). Fish collagen peptide (molecular weight 2000–5000 Da) and bovine collagen peptide (molecular weight < 3000 Da) were purchased from Xi’an Gawen Biotechnology Co., Ltd. (Xi’an, China).

### 2.2. Synthesis of Composite Materials

Firstly, HAp powder was prepared via wet precipitation method. Synthesis was performed at room temperature and at constant stirring while ammonium phosphate monobasic (0.36 mol/L) and calcium nitrate tetrahydrate (0.60 mol/L) were used as reagents. The calcium nitrate solution was added dropwise (1 drop/s) into the ammonium phosphate solution while maintaining the alkaline pH of the reaction mixture (pH > 10) by adding ammonia water. pH of the reaction mixture was controlled in a continuous manner via a multifunctional CX-701 pH-meter (Elmetron, Zabrze, Poland). After the reaction, the mixture obtained was allowed to sediment for 24 h. The sediment was washed with distilled water to neutral pH, lyophilized (parameters: T = −50 °C, p = 0.07 mbar, t = 24 h) and used in further works for preparation of polymer-ceramic composites.

PVP/PVA-based composite materials containing HAp were prepared according to procedure described previously in [36]. Briefly, the aqueous solutions of polymers—PVA and PVP—were mixed with a ceramic phase (HAp) and selected collagen, i.e., fish collagen or bovine collagen. Next, an appropriate amount of crosslinking agent PEGDA with an average molecular weight of either 575 g/mol or 700 g/mol and photoinitiator (2-hydroxy-2-methylpropiophenone) was added. The whole mixtures were placed in vessels of the selected shapes and polymerized under UV lamp (EMITA VP-60, power: 180 W, λ = 320 nm; Famed, Lodz, Poland). The process of photopolymerization was performed for 120 s. Detailed compositions of obtained polymer-ceramic composites are given in Table 1.

**Figure 1:**
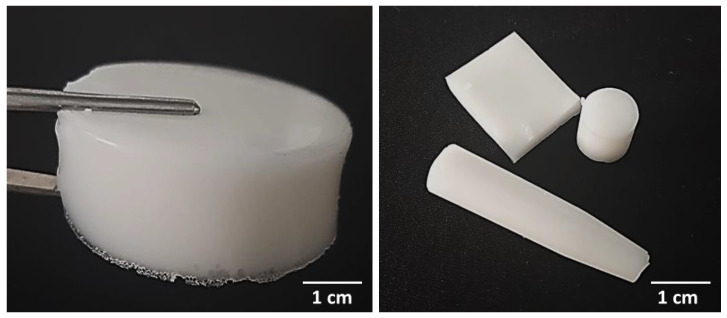
Various shapes of prepared composite materials—sample 575_HAp/Coll-fish (**left**) and samples 700_HAp/Coll-bov (**right**).

The use of vessels with various shapes allowed to obtain composites of selected shapes and sizes; prepared materials are presented in Figure 1.

Prepared polymer-ceramic composites were subsequently subjected to physicochemical evaluation aimed at determining their properties such as swelling ability, surface hydrophilicity or roughness.

### 2.3. Characterization of the Chemical Structure of Composite Materials via Fourier Transform Infrared (FT-IR) Spectroscopy

FT-IR spectroscopy was performed to identify the functional groups present in the structures of tested materials while the main attention was paid to group characteristics for collagen. The analysis was conducted for samples without this protein as well as for samples modified with collagen and prepared using crosslinking agents with different average molecular weights. The investigations were carried out at room temperature and using a Thermo Scientific Nicolet iS5 FT-IR spectrophotometer equipped additionally with iD7 ATR (Attenuated Total Reflectance, Loughborough, UK) accessory. FT-IR spectra were recorded within the wavenumber range 4000–500 cm^−1^ (32 scans, resolution 4.0 cm^−1^).

### 2.4. Evaluation of the Swelling Properties of Composite Materials

Subsequent study concerned the evaluation of swelling ability of the composites. Considering intended application of developed composites for biomedical purposes, it is significant to determine their behavior in liquids simulating environments occurring in the human body. Thus, the investigations were performed using such liquids as SBF (simulated body fluid), Ringer liquid (isotonic to human blood) and distilled water as a reference liquid. In order to determine this property, prepared materials were dried at 37 °C for 24 h, weighed and placed in 50 mL of the mentioned solutions. After selected time periods—i.e., 1 h, 24 h, 48 h and 72 h—samples were separated from the solutions, an excess of unbound water was removed using a paper towel and samples were weighed again. The swelling ability defined using a swelling ratio *α* was determined via the following Equation (1):

(1) $$\alpha =\frac{\left(m-m_{0}\right)}{m_{0}}$$

where:

*α*—swelling ratio, g/g; *m*—mass of swollen sample, g/g; and *m*_0_—mass of dry sample (before the study), g.

The study was performed for all prepared samples, i.e., both for unmodified materials and materials containing collagen.

### 2.5. Incubation Studies

The study was performed to verify the interaction between simulated physiological liquids and prepared composite materials during their long-term immersion in such environments. Samples of the composites (weighing approx. 1.0 g) were placed in 50 mL of selected liquids (the same liquids were used as in the case of swelling studies, i.e., SBF, Ringer liquid and distilled water) whose pH values were verified every two days. The study was conducted for 14 days while samples were incubated at 36.6 °C so at temperature of human body. Both samples with and without collagen were subjected to the research.

### 2.6. Evaluation of the Wettability of Composites

Wettability of prepared materials was measured by a sessile drop method followed by Drop Shape Analysis system—DSA 10Mk2, Kruss, Germany. Contact angle was determined for polar solvent which was ultra-high quality (UHQ) water (Purelab UHQ, Elga) of resistivity 18 MΩ/cm, and non-polar diodomethane (Sigma Aldrich). Surface free energy was calculated according to the Owens, Wendt, Rabel and Kaelble (OWRK) approach.

The mean contact angle values and deviation were determined from three repetitions measurements at different spots on the sample.

### 2.7. Assessment of Roughness of Composites

The surface roughness was measured using a stylus contact profilometer TALYSURF6 (Taylor Hobson, San Francisco, CA, USA) Surface roughness was measured according to ISO 4287 [37] with contact stylus acquisition mode in a line distance of 4 mm. The roughness profile including parameters Ra (arithmetic mean of the departures), Rq (kurtosis) and Rsk (skewness) numerically describe the topography of the measured surfaces. The mean value and deviation was determined from at least three repetition measurements at different spots on the sample. The TalyMap Platinum software (Taylor Hobson, San Francisco, CA, USA) was used for 3D topography analysis and export of the surface images.

### 2.8. Statistical Analysis of the Results of the Investigations

The results of the investigations were subjected to the statistical analysis performed via the two-way analysis of variance (ANOVA) while alpha value 5% was applied. All measurements were carried out three times (n = 3) and are presented as an average value with the standard deviation (SD).

## 3. Results and Discussion

### 3.1. Results of FT-IR Spectroscopy

**Figure 2:**
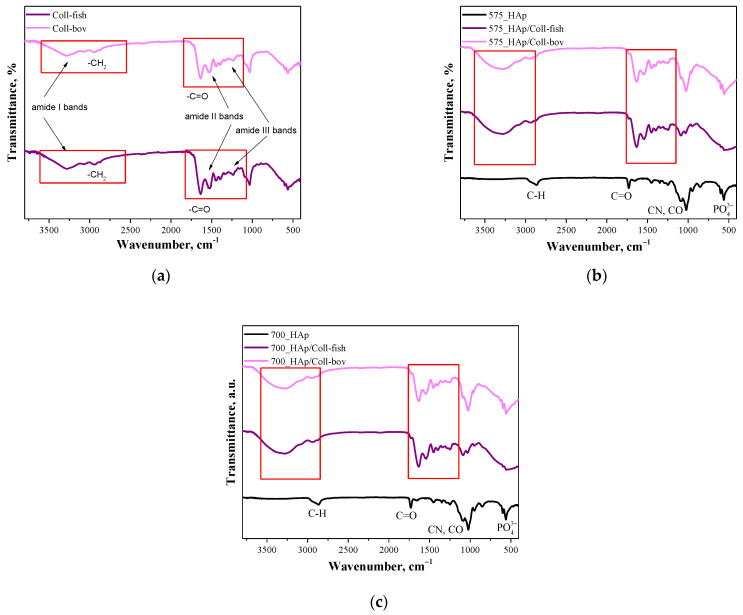
FT-IR spectra of both used collagens—fish collagen (Coll-fish) and bovine collagen (Coll-bov) (**a**); composites prepared using PEGDA 575 (**b**) and composites prepared using PEGDA 700 (**c**).

Below in Figure 2 FT-IR, spectra of unmodified and modified composite materials are presented. The study was performed both for samples containing fish collagen (Coll-fish) and bovine collagen (Coll-bov).

In Figure 2a FT-IR, spectra of fish collagen and bovine collagen are presented. In the case of both samples, absorption bands characteristic for functional groups present in the structure of the mentioned proteins were identified. The absorption bands at 3284 cm^−1^ have been assigned to amide I bands corresponding to hydrogen bonds between NH group and carbonyl group of the peptide chain. Next, the absorption bands at approx. 2939 cm^−1^ deriving probably from the asymmetrical stretching vibrations of CH_2_ characteristic for amide B bands were identified. In turn, bands within the wavenumber range 1700–1600 cm^−1^ which can be attributed to the stretching vibrations of C=O from peptide bonds (determined also as amide I bands) confirmed the occurrence of secondary structure characteristic for proteins. Moreover, the absorption bands deriving also from amide II and amide II may also be observed on analyzed FT-IR spectra. Next, the absorption bands within the range 1570–1470 cm^−1^ characteristic for amide II which may be assigned for N–H bending vibrations coupled with C–N stretching vibration, were observed; while at 1350–1250 cm^−1^ (amide III), absorption bands characteristic for stretching vibrations of C–H group were identified. Bands marked in Figure 2a were identified analogously as in other works [38,39,40,41]. Importantly, the absorption bands most characteristic for collagen identified also on FT-IR spectra of polymer-ceramic composites containing this protein (fish or bovine origin) have been marked with a red frame in Figure 2a–c.

### 3.2. Studies on Swelling Capacity of Composite Materials

**Figure 3:**
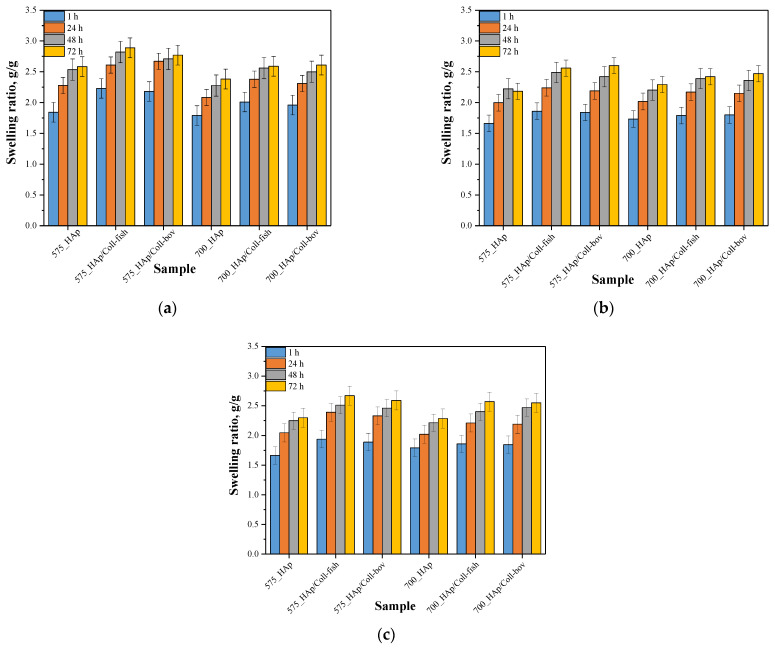
Results of swelling studies of composite materials in distilled water (**a**), SBF (**b**) and Ringer liquid (**c**) (n—number of repetitions, n = 3).

Next, investigations were aimed at determining swelling capacity of composite materials while the main purpose of the study was to verify the potential difference in this property between unmodified materials and materials modified with collagen. Importantly, the impact of the average molecular weight of the crosslinking agent used during the synthesis of composites on their swelling sorption was also discussed. Results of performed studies are presented in Figure 3. In Table 2, the statistical analysis of obtained data is presented.

One of the basic properties of hydrogel materials is their ability to absorb liquids without permanent loss of shape and deterioration of mechanical properties. These abilities result mainly from the hydration of such hydrophilic functional groups as –OH, −COOH or –CONH_2_ occurring in the structure of the polymers and also are due to the presence of capillary forces. Importantly, during such a sorption these materials do not dissolve, which is due to numerous covalent or hydrogen bonds or electrostatic interactions between polymer chains.

Based on the results of performed swelling investigations, it may be concluded that all tested materials show swelling properties. The highest swelling ratios were calculated for samples swelling in distilled water while the sorption in Ringer liquid and in SBF was significantly lower. For example, sample 575_HAp after 1 h swelling showed α = 1.84 g/g in distilled water, α = 1.67 g/g in Ringer liquid and α = 1.66 g/g in SBF. Such results result from the fact that the swelling process depends strictly both on the composition of tested sample and the composition of the liquid in which sorption takes place. Lower values of swelling ratios in SBF and Ringer liquid result probably from the occurrence of divalent ions in these liquids which may, in turn, affect the formation of additional crosslinks between polymer chains thus increasing the crosslinking density of the polymer. This, in turn, leads to a decrease of free spaces between polymer chains available for absorbed liquid and finally to lower swelling properties of samples in such liquids. In the case of distilled water, where any additional ions do not occur, the above-described phenomenon concerning the formation of additional crosslinks does not take place so there is more free space between polymer chains for absorbed liquid.

Moreover, it was observed that all samples prepared using a crosslinking agent with an average molecular weight 575 g/mol (PEGDA 575) showed higher swelling ability than materials obtained using PEGDA 700. The observed dependence is related to a different structure of crosslinked materials depending on the crosslinking agent used. The highest differences were observed during first 24 h of the swelling. Analogous results were presented in other works [36]. The use of the crosslinking agent PEGDA 575 resulted in the synthesis of polymer matrices consisting of shorter polymer chains compared to the chains occurring in the structure of materials obtained using PEGDA 700. Such a difference affects the swelling properties of tested materials—shorter polymer chains resulted in a formation of more developed and porous structure which may absorb more liquids. Thus, the highest differences in swelling ratios of composites obtained using different crosslinking agents were observed between values calculated after 1 h and after 24 h when the material absorbs liquids mainly on the surface. During the next hours of swelling, a penetration of liquids into the interior of analyzed polymer samples takes place, therefore differences between swelling ratios after 48 h and 72 h compared to α after 24 h were slight. On the other hand, any significant impact of the type of the collagen used on the swelling properties of modified composite materials was not observed. Similar observations concerning the sorption capacity of the materials modified with collagens of various origin were presented by Ghodbane et al. [42]. Nonetheless, the differences between unmodified samples and samples modified with collagen were observed. Both introduction of fish collagen and bovine collagen into the composite materials resulted in an increase in their swelling properties. In general, collagen is responsible for water binding in tissues. Thus, its ability of water sorption translates into the highest swelling ability of composites modified with this protein compared to unmodified materials. These results are consistent with results presented by Bai et al. who stated that as a result of the modification of materials with collagen these materials had a higher amount of hydrophilic functional groups which may interact with water [43]. As a result, materials with collagen show a higher swelling capacity that was also reported in investigations presented here.

### 3.3. Results of Incubation of Composite Materials in Selected Simulated Physiological Liquids

In Table 3, Table 4 and Table 5, results of 14-day incubation of composite materials are presented. During the study, the change in pH was determined as a function of time while the measurements were also performed for incubation liquids without samples (reference measurements).

Based on the above presented results, it may be concluded that in the case of all tested samples, pH values measured in the course of their incubation varied, but only slightly. Rapid jumps in pH values could indicate the degradation of polymer matrices or the release of potential unreacted reagents such as crosslinking agent or photoinitiator from their interior. In the case of tested composite materials, such changes were not observed, which, in turn, may be evidence of the preparation of properly-crosslinked materials which show biocompatibility with tested environments. The only big difference (compared to the other results) was observed in the case of samples modified with collagen. This was probably related to the method of its preparation. The collagen hydrolysates introduced into the polymer matrices were obtained as a result of an enzymatic hydrolysis performed in an acidic environment which, in turn, may translate into the mentioned pH decrease during the incubation of such modified materials. However, these changes were slight and, importantly, the least noticeable for samples immersed in SBF, which may probably be evidence of well buffering properties of prepared composites in this liquid.

### 3.4. Results of Investigations on Wettability of Composites

**Figure 4:**
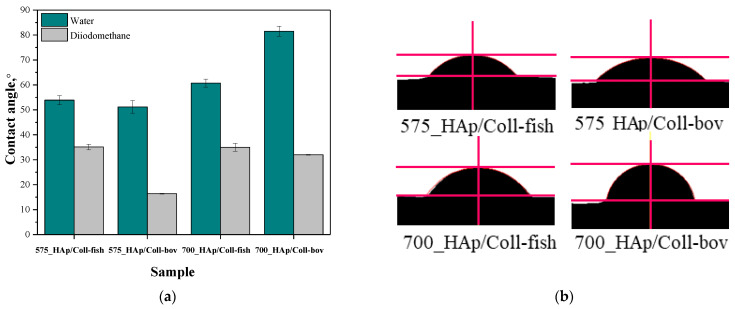
Contact angles of composite materials modified with collagen (fish or bovine one) determined both for distilled water and diiodomethane (**a**) and example images of distilled water-wetted composites (**b**) (n—number of repetitions, n = 3).

In order to verify the hydrophilic/hydrophobic nature of the surface of prepared composite materials, the values of their contact angles were determined. The study was conducted using two measuring liquids, i.e., distilled water and diiodomethane. Results of the research are shown in Figure 4 while in Table 6 the statistical analysis of obtained data is presented.

Furthermore, the analysis performed using two mentioned measuring liquids, i.e., both polar and non-polar one, allowed to determine the surface free energies of analyzed composite materials, whose values are presented in Table 7.

Proper dental orthopedic implantation depends strictly on the osteointegration degree, i.e., on the possibility of formation of stable connection between the implant and the tissue. This, in turn, depends on many factors including e.g., the physicochemistry of the implant surface. One of the most important parameters of such a surface is its hydrophilicity. Thus, it was important to perform investigations aimed at determining the contact angles of obtained composite materials which, in turn, allowed to define the hydrophilic or hydrophobic nature of their surfaces. For example, it was demonstrated that hydrophilic surfaces affect the formation of the environment conducive to bone growth [44]. It is assumed that the mentioned dependency is related to the rapid spread of serum on the hydrophilic surface of the implant which, in turn, provides a good substrate for bioactive substances [45]. These substances may significantly affect the early adhesion of cells, their proliferation and differentiation [46,47]. The materials whose contact angle with polar liquid is less than 90° are defined as hydrophilic. Thus, it may be concluded that all tested composites are hydrophilic in nature. This is a significant advantage of developed materials when considering their potential use for biomedical use including tissue engineering.

In the case of unmodified samples, i.e., 575_HAp and 700_HAP, determining the contact angle was not possible. The drops of the measuring liquid spread over the surface of these material at the very first moment of the measurement. Thus, the images showing the first contact of a drop with unmodified materials were not recorded which, in turn, made it impossible to determine the contact angles of these samples.

In the case of the rest of tested samples, it may be concluded that composites prepared using PEGDA 575 showed lower contact angles than samples obtained using PEGDA 700, indicating better surface wettability. Values of contact angles of samples synthesized using PEGDA 700 were significantly higher, which indicated a less hydrophilic nature of their surfaces. Here, due to the use of the crosslinking agent with a higher average molecular weight during the synthesis, prepared materials consisted of long polymer chains. As a result, functional groups deriving from collagen as well as functional groups from the structure of PVP and PVA responsible for interactions with polar measuring liquid (distilled water) may be “embedded” between these long chains (“trapped” inside the polymer matrix). On the other hand, the polymer matrix obtained using PEGDA 575 consisted of short polymer chains, thereby the mentioned hydrophilic functional groups may be more exposed and thus more available for interactions with measuring liquid via e.g., hydrogen bonds.

### 3.5. Results of the Evaluation of the Surface Roughness of Composite Materials

The roughness of tested materials is another important parameter affecting the adhesion and growth of cells, therefore the next investigations concerned the determination of parameters describing the surface roughness. The study was performed via the profile method according to the ISO 4287 standard [37]. Values characterizing the roughness of tested materials are presented in Table 8, and statistical analysis of obtained data is presented in Table 9.

**Figure 5:**
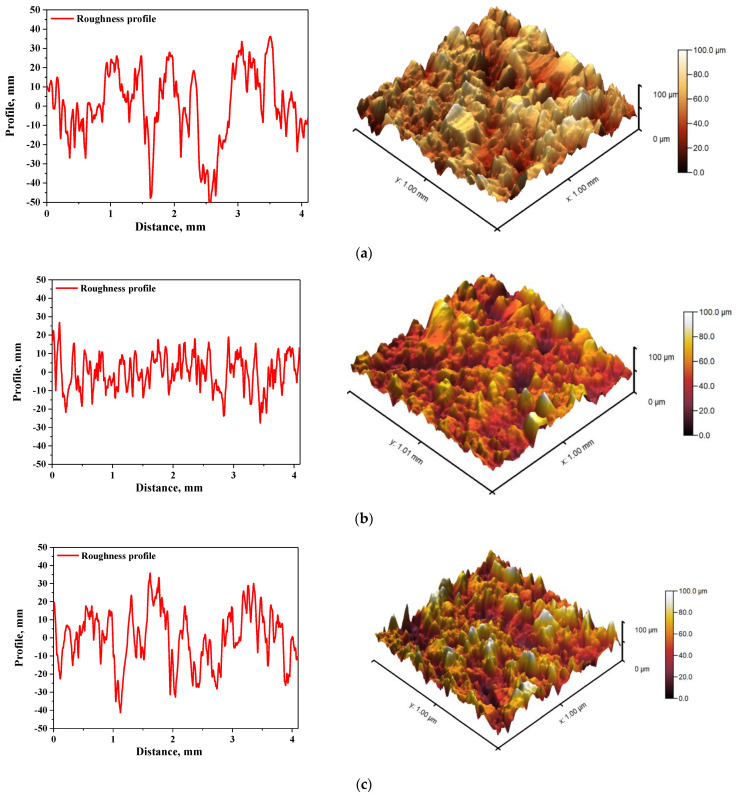
The roughness profile (left) and 3D image of sample 575_HAp (**a**), 575_HAp/Coll-fish (**b**) and 575_HAp/Coll-bov (**c**).

**Figure 6:**
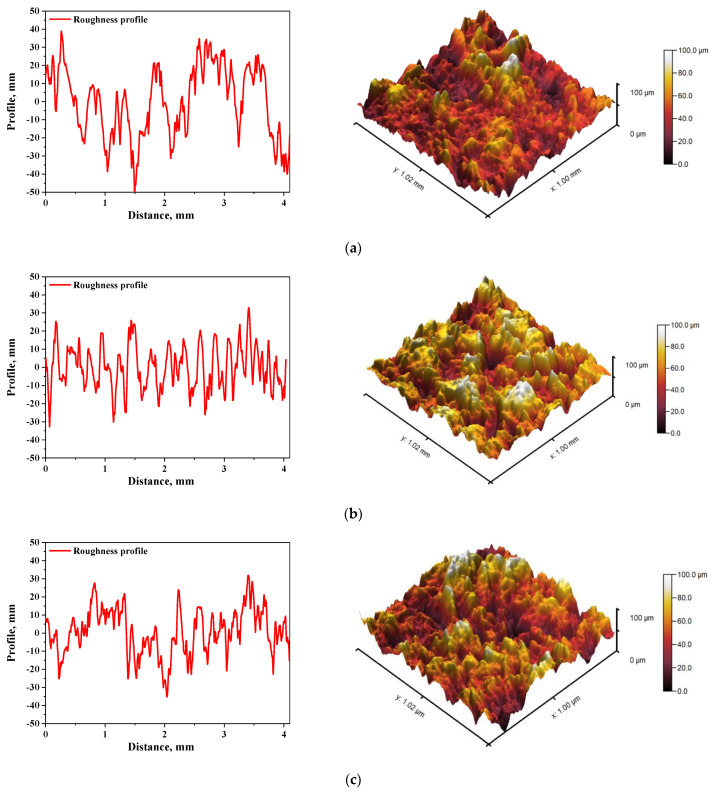
The roughness profile (left) and 3D image of sample 700_HAp (**a**), 700_HAp/Coll-fish (**b**) and 700_HAp/Coll-bov (**c**).

In Figure 5 and Figure 6, roughness profiles and surface topography of analyzed materials are shown.

The roughness and the surface topography of composite materials designed for application in implantology and tissue regeneration are one of the most important factors which may promote the osteointegration. The roughness of the surface had a great impact on the potential interactions between newly formed bone tissue and the implant. The proliferation of cell as well as the formation of extracellular matrix at the interface between the implant and the healthy tissue is a key aspect in the bone reconstruction process. For example, in previous studies it was proved that rough and bioactive surface of the implant may provide an improved adhesion and proliferation of human osteoblasts [48]. In the case of all tested materials, roughness parameter R_a_ was within the range 7.98–9.63 µm, which indicated a relatively high roughness of analyzed composites. This, in combination with the fact that analyzed materials were modified with a ceramic phase, i.e., hydroxyapatite, which provides osteoinductive and osteoconductive properties, allowed to conclude that developed materials showed properties conducive to bone tissue regeneration due to the roughness of their surface and simultaneous bioactivity provided via a presence of a ceramic phase.

## 4. Conclusions

Spectroscopic analysis confirmed the occurrence of absorption bands characteristic for the modifier used, i.e., collagen. This, in turn, confirmed the effectiveness of the synthesis methodology applied which led to obtaining modified composites.

Next, it was proved that all tested composite materials showed swelling properties in simulated physiological liquids while the highest sorption was observed in distilled water (swelling ratio α = 2.0 g/g after 1 h). Swelling in SBF and Ringer liquid was adequately 11.0% and 8.5% lower, which was a result of the presence of numerous ions in these solutions which may increase the crosslinking density of such materials.

Impact of the modification of composites on their physicochemical properties was demonstrated. It was proved that modification of polymer–ceramic composites with collagen resulted in an increase of swelling ratios by 0.5–1.0 g/g compared to unmodified composites. Next, considering the results of wettability investigations, it was demonstrated that values of contact angles determined for polymer–ceramic composites modified with collagen were lower than 90°, which indicated the hydrophilic nature of the surfaces of developed materials. Furthermore, performed statistical analysis proved that the both the type of the crosslinking agent used and the type of collagen had a statistically significant impact on such properties as wettability and swelling ability in selected conditions.

During 14-day incubation of composites in simulated physiological liquids, any rapid pH changes were not observed. Such changes might indicate the potential degradation of immersed liquids.

In the case of studies on roughness of composite materials, any statistically significant impact of both the average molecular weight of the crosslinking agent used and the type of collagen introduced into the polymer matrix, was not demonstrated. All composites showed similar surface roughness, which was due to a ceramic phase (hydroxyapatite) present in all tested materials.

Considering such advantages of developed polymer–ceramic composites modified with collagen as their quick and simple synthesis, a possibility of the preparation of their various shapes and sizes depending on the vessel in which the polymerization is performed as well as their properties desirable in viewpoint of bone tissue regeneration, it may be concluded that developed materials showed great application potential and should be subjected to more advanced experiments.

## Figures and Tables

**Table 1:** Compositions of polymer-ceramic composites.

15% PVP, mL	5% PVA, mL	HAp, wt.%	Photoinitiator, mL	Crosslinking Agent, mL	Fish Collagen, wt.%	Bovine Collagen, wt.%	Sample Name
3	7	10	0.05	2.0 (575 g/mol)	-	-	575_HAp
					30	-	575_HAp/Coll-fish
					-	30	575_HAp/Coll-bov
				2.0 (700 g/mol)	-	-	700_HAp
					30	-	700_HAp/Coll-fish
					-	30	700_HAp/Coll-bov

**Table 2:** Statistical analysis of obtained data based on the two-way analysis of variance (ANOVA) with repetitions.

Independent Variable	Study	*p* *
Type of crosslinking agent	1 h swelling in distilled water	1.52923 × 10^−15^
Type of collagen		2.96059 × 10^−14^
Type of crosslinking agent	72 h swelling in distilled water	8.39488 × 10^−15^
Type of collagen		5.92119 × 10^−14^
Type of crosslinking agent	1 h swelling in SBF	0.42331
Type of collagen		0.42271
Type of crosslinking agent	72 h swelling in SBF	8.12234 × 10^−15^
Type of collagen		7.31011 × 10^−14^
Type of crosslinking agent	1 h swelling in Ringer liquid	3.97822 × 10^−14^
Type of collagen		1.54037 × 10^−13^
Type of crosslinking agent	72 h swelling in Ringer liquid	6.04203 × 10^−14^
Type of collagen		1.18424 × 10^−13^

* *p* indicates the statistical significance calculated using the two-way analysis of variance (ANOVA).

**Table 3:** Results of incubation in distilled water.

Sample	pH Value							
	Day 0	Day 2	Day 4	Day 6	Day 8	Day 10	Day 12	Day 14
Distilled water	7.218 ± 0.216	7.114 ± 0.211	7.015 ± 0.213	7.054 ± 0.209	7.177 ± 0.214	6.912 ± 0.211	7.036 ± 0.216	6.991 ± 0.208
575_HAp	7.132 ± 0.215	7.141 ± 0.212	7.053 ± 0.209	6.990 ± 0.210	7.012 ± 0.215	6.984 ± 0.208	7.013 ± 0.213	7.181 ± 0.214
700_HAp	7.041 ± 0.211	7.028 ± 0.208	6.986 ± 0.210	6.944 ± 0.208	6.972 ± 0.211	6.954 ± 0.209	7.021 ± 0.208	7.047 ± 0.211
575_HAp/Col-fish	7.183 ± 0.215	6.604 ± 0.198	6.694 ± 0.201	6.756 ± 0.205	6.833 ± 0.202	6.757 ± 0.199	6.655 ± 0.203	6.842 ± 0.201
700_HAp/Col-fish	7.144 ± 0.214	6.557 ± 0.194	6.645 ± 0.201	6.723 ± 0.192	6.782 ± 0.203	6.710 ± 0.198	6.681 ± 0.203	6.796 ± 0.196
575_HAp/Col-bov	7.164 ± 0.215	6.564 ± 0.198	6.656 ± 0.197	6.715 ± 0.202	6.793 ± 0.204	6.511 ± 0.195	6.612 ± 0.197	6.809 ± 0.203
700_HAp/Col-bov	7.148 ± 0.214	6.534 ± 0.202	6.625 ± 0.198	6.687 ± 0.203	6.764 ± 0.195	6.486 ± 0.197	6.582 ± 0.194	6.773 ± 0.200

**Table 4:** Results of incubation in SBF.

Sample	pH Value							
	Day 0	Day 2	Day 4	Day 6	Day 8	Day 10	Day 12	Day 14
SBF	7.485 ± 0.222	7.491 ± 0.226	7.542 ± 0.225	7.493 ± 0.224	7.438 ± 0.224	7.501 ± 0.227	7.493 ± 0.222	7.570 ± 0.225
575_HAp	7.526 ± 0.226	7.466 ± 0.224	7.546 ± 0.223	7.496 ± 0.226	7.406 ± 0.227	7.566 ± 0.225	7.516 ± 0.223	7.636 ± 0.224
700_HAp	7.584 ± 0.227	7.484 ± 0.223	7.434 ± 0.222	7.444 ± 0.222	7.451 ± 0.223	7.474 ± 0.225	7.474 ± 0.224	7.444 ± 0.223
575_HAp/Col-fish	7.576 ± 0.228	7.455 ± 0.226	7.425 ± 0.227	7.435 ± 0.224	7.505 ± 0.229	7.465 ± 0.227	7.465 ± 0.226	7.435 ± 0.226
700_HAp/Col-fish	7.585 ± 0.224	7.484 ± 0.227	7.633 ± 0.223	7.646 ± 0.224	7.511 ± 0.226	7.572 ± 0.227	7.572 ± 0.226	7.546 ± 0.225
575_HAp/Col-bov	7.557 ± 0.226	7.413 ± 0.223	7.443 ± 0.223	7.453 ± 0.224	7.423 ± 0.227	7.483 ± 0.226	7.483 ± 0.227	7.453 ± 0.225
700_HAp/Col-bov	7.694 ± 0.231	7.496 ± 0.229	7.423 ± 0.227	7.431 ± 0.226	7.405 ± 0.224	7.461 ± 0.225	7.463 ± 0.227	7.434 ± 0.225

**Table 5:** Results of incubation in Ringer solution.

Sample	pH Value							
	Day 0	Day 2	Day 4	Day 6	Day 8	Day 10	Day 12	Day 14
Ringer solution	5.749 ± 0.172	5.881 ± 0.176	5.941 ± 0.174	5.820 ± 0.175	5.842 ± 0.173	5.841 ± 0.172	5.850 ± 0.176	6.051 ± 0.181
575_HAp	5.654 ± 0.169	5.794 ± 0.173	5.854 ± 0.175	5.834 ± 0.175	5.854 ± 0.181	5.754 ± 0.176	5.864 ± 0.174	6.064 ± 0.176
700_HAp	5.751 ± 0.169	5.891 ± 0.164	5.851 ± 0.166	5.831 ± 0.167	5.851 ± 0.164	5.751 ± 0.166	5.861 ± 0.169	6.061 ± 0.170
575_HAp/Col-fish	5.634 ± 0.171	5.474 ± 0.175	5.534 ± 0.172	5.474 ± 0.181	5.434 ± 0.174	5.534 ± 0.175	5.544 ± 0.174	5.584 ± 0.176
700_HAp/Col-fish	5.641 ± 0.169	5.481 ± 0.164	5.541 ± 0.166	5.421 ± 0.167	5.441 ± 0.164	5.541 ± 0.169	5.551 ± 0.167	5.451 ± 0.168
575_HAp/Col-bov	5.625 ± 0.167	5.465 ± 0.168	5.425 ± 0.165	5.405 ± 0.162	5.425 ± 0.166	5.525 ± 0.167	5.475 ± 0.164	5.435 ± 0.169
700_HAp/Col-bov	5.621 ± 0.168	5.561 ± 0.165	5.621 ± 0.168	5.501 ± 0.166	5.421 ± 0.168	5.521 ± 0.169	5.531 ± 0.171	5.541 ± 0.167

**Table 6:** Statistical analysis of obtained data based on the two-way analysis of variance (ANOVA) with repetitions.

Independent Variable	Measuring Liquid	*p* *
Type of crosslinking agent	Distilled water	0.00157
Type of collagen		0.01485
Type of crosslinking agent	Diiodomethane	4.85299 × 10^−4^
Type of collagen		0.00591

* *p* indicates the statistical significance calculated using the two-way analysis of variance (ANOVA).

**Table 7:** Surface free energies determined for tested composites.

Sample *	Surface Free Energy		
	Polar, mJ/m^2^	Dispersive, mJ/m^2^	Total Free Energy, mJ/m^2^
575_HAp/Coll-fish	21.28	26.59	47.87
575_HAp/Coll-bov	18.71	33.74	52.45
700_HAp/Coll-fish	14.90	29.74	44.64
700_HAp/Coll-bov	1.40	43.66	45.06

* for samples HAp_ 575 and HAp_ 700 values of contact angles could not be determined.

**Table 8:** Roughness parameters according to ISO 4287 standard [37].

Sample Name	R_a_, µm	R_q_, µm	R_sk_, µm
575_HAp	8.18 ± 1.36	9.87 ± 1.79	−0.09 ± 0.17
575_HAp/Coll-fish	9.63 ± 0.06	11.71 ± 0.06	−0.07 ± 0.03
575_HAp/Coll-bov	9.02 ± 0.53	11.15 ± 0.38	−0.02 ± 0.32
700_HAp	8.42 ± 1.49	10.15 ± 1.56	−0.11 ± 0.31
700_HAp/Coll-fish	9.17 ± 0.96	11.23 ± 1.18	−0.11 ± 0.23
700_HAp/Coll-bov	7.98 ± 0.68	10.45 ± 1.57	−0.12 ± 0.47

**Table 9:** Statistical analysis via the two-way analysis of variance (ANOVA) with repetitions.

Independent Variable	*p* *		
	R_a_	R_q_	R_sk_
Type of crosslinking agent	0.08072	0.19422	0.89284
Type of collagen	0.21614	0.48395	0.94069
Addition of Coll-fish (compared to unmodified material)	0.25956	0.26533	0.95593
Addition of Coll-bov (compared to unmodified material)	0.43402	0.04523	0.86937

* *p* indicates the statistical significance calculated using the two-way analysis of variance (ANOVA).

## Data Availability

The data presented in this study are available on request from the corresponding authors.
